# Iconic logic: the visual art of drawing the right conclusion

**DOI:** 10.3389/fpsyg.2024.1368989

**Published:** 2024-06-07

**Authors:** Peter Kramer

**Affiliations:** Department of General Psychology, University of Padua, Padua, Italy

**Keywords:** existential graph, concept diagram, iconic logic, logical graph, iconic mathematics, diametric theory, social exchange, reasoning ability

## Abstract

Most people, evidence suggests, have a hard time thinking straight. *Symbolic logic* is a tool that can help remedy this problem. Unfortunately, it is highly abstract and uses symbols whose meanings rely on unintuitive arbitrary conventions. Without sacrificing rigor, *iconic logic* is more concrete and uses icons that resemble what they stand for and whose meanings are thus easier to picture, process, and remember. Here I review and critique iconic *existential graphs* and *concept diagrams*—the former link iconic logic to iconic mathematics; the latter expand popular Euler or Venn diagrams and have, to some degree, been empirically investigated for user-friendliness. I lay out how expertise in perception, cognition, and genetics can inform and improve such empirical research to help make iconic logic more ergonomic. After all, logic is a tool, and tools should not only suit their use but also their user.

*No, no, you are not thinking, you are just being logical.*

—Niels Bohr (Frisch, 1979).

## 1 Introduction

### 1.1 Toward a rigorous but more user-friendly logic

Formal logic offers rules for how to reason in a watertight, step-by-step manner to derive indisputable conclusions from a given set of starting assumptions. It is a great tool but typically not very user friendly. Here, I present a review of attempts to make it more ergonomic without sacrificing its rigor. I discuss two important solutions: *existential graphs* (particularly interesting because a precursor of them inspired a more general “iconic mathematics”) and *concept diagrams* (particularly interesting because they inspired behavioral experiments). To my knowledge, no behavioral scientists have been involved in designing these behavioral experiments, but I argue that it would have been good—and still is—to get experts on perception and cognition on board. By uncovering the logician’s typical psychological profile, I explain why most logicians seem to underappreciate the need for a more ergonomic kind of logic, just like most mathematicians seem to underappreciate the need for a more ergonomic mathematics (Kramer, 2022). I refer to gene expression to explain why many otherwise intelligent people are poor logicians and why, particularly for them, iconic logic is a better tool than is symbolic logic.

### 1.2 Icons versus symbols

Logic is expressed with the help of signs. A sign is something that stands for something else. According to Charles Sanders Peirce, one of the founders of semiotics (the study of signs) a sign is either a token or a type of tokens (Nöth, 1990; Peirce, 1992). A token stands for an instance (e.g., a particular woman) of a type of tokens (the set of women in general). According to Peirce, a sign is also either an index, icon, or symbol (Nöth, 1990; Peirce, 1992). An index directs attention to what it stands for, like smoke to fire or a finger to what it points at. An icon resembles what it stands for, like a picture of a train resembles a train, the word “splash” the sound of a splash, and six dots on a die a quantity of six. A symbol refers to what it stands for by an arbitrary convention or chance association, like for no apparent reason “+” means “plus,” “∴” means “therefore,” and “6” and “six” a quantity of six again.

The extent to which a sign is an index, icon, or symbol is a matter of degree. The more a sign is a symbol, the less intuitive, harder to remember, and less reader-friendly it is. Ironically, rather than on icons or indices, formal logic relies most heavily on precisely this kind of signs. Many symbols, moreover, are assigned only a temporary meaning that can change from context to context. The letters *x* and *y*, for example, may stand for “ewes” and “rams” in one context but for, say, “midgets” and “elves” in another. Especially when there are many of them, symbols burden readers’ memory more than do other signs. Symbolic logic is therefore typically only used by experts with, as I shall argue later, particularly strong memories of exactly the right type.

Iconic logic offers an alternative to symbolic logic. Without sacrificing rigor, it relies less on symbols and more on icons. Iconic logic still is partly symbolic. Yet just like the hallmark of the chocolate bar is that it has chocolate, even though it also has other ingredients, the hallmark of iconic logic is that it is more iconic than symbolic logic is, even though it is also to some extent symbolic (Stjernfelt, 2007; Kralemann and Lattmann, 2013; Stjernfelt, 2022). Calling chocolate bars “candy bars” is accurate but misses what is important about them. Likewise, calling iconic logic “graphical logic” or “diagrammatic logic” also misses the point. It is its superior iconicity that makes iconic logic worth its name (Shin, 2002; Bricken, 2019a) and more intuitive than symbolic logic can ever be. The icons that are the easiest to process, picture in one’s mind, and remember, represent only the essential features or gist of what they stand for and leave out irrelevant details (Kramer, 2023). So, although a photo of a train resembles a train more than a highly simplified drawing of it does, iconic logic uses the latter, minimalist type of icons rather than the former, more complex ones.

**Figure 1:**
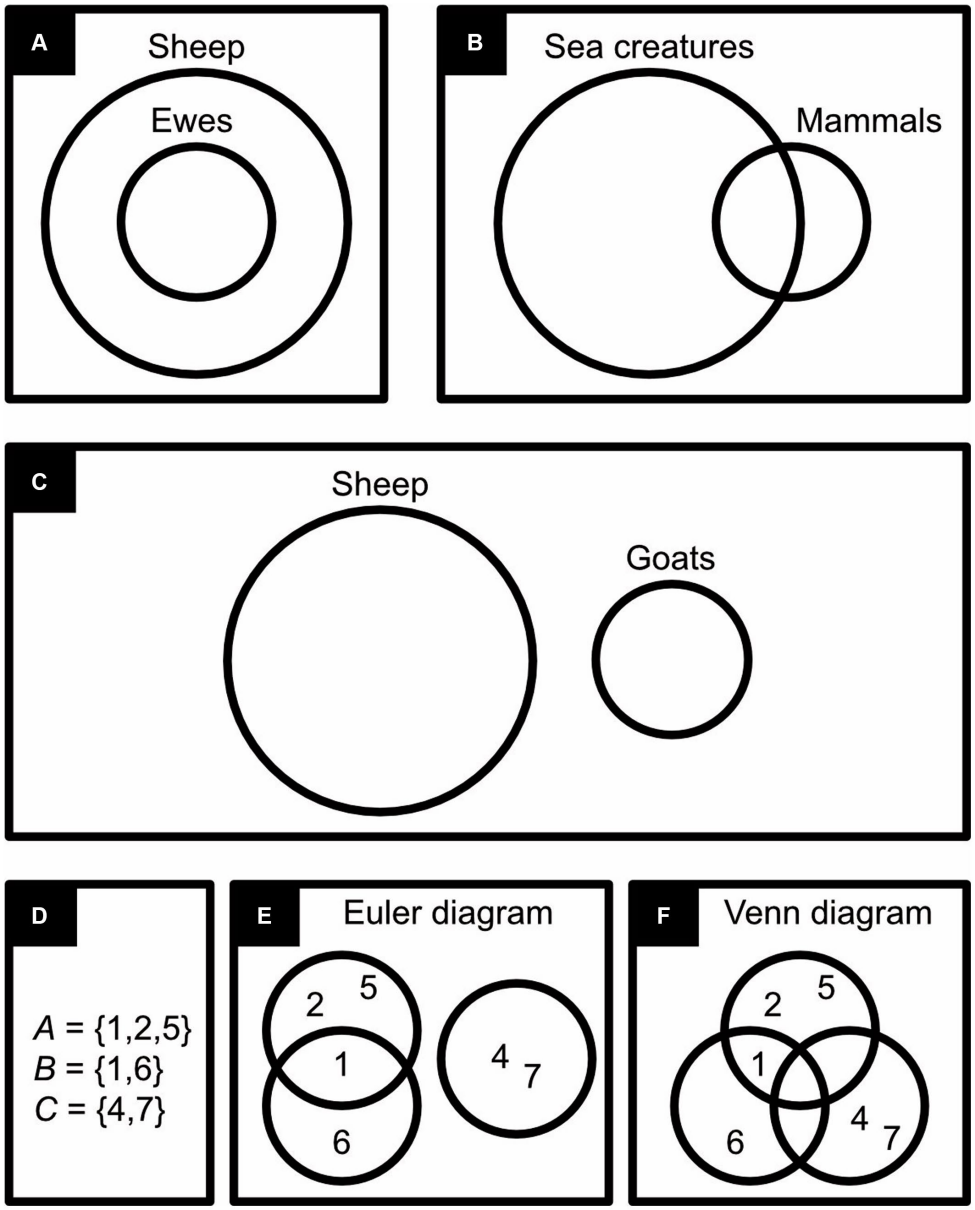
Euler and Venn diagrams. The first three diagrams express: **(A)** “All ewes are sheep”. **(B)** “Some sea creatures are mammals,” **(C)** “Things can be sheep or goats but not both”. The last three diagrams represent three sets of numbers in symbolic notation **(D)** in an Euler diagram **(E)** and in a Venn diagram **(F)**.

To some extent, symbolic logic is also iconic. For example, in P ⊂ Q, by convention, P and Q represent sets, but ⊂ is narrow on the extreme left and wide on the right, which vaguely suggests that P is part of something bigger, that P is a subset of Q. Symbolic logic is also somewhat iconic where it presents similar things in similar form, like when it presents variables in lowercase but predicates (properties and relationships) in uppercase (Stjernfelt, 2007; Kralemann and Lattmann, 2013; Stjernfelt, 2022). Euler diagrams (Euler and Brewster, 1833), however, represent sets and basic relationships between sets in a much more iconic and thus intuitive way. In such diagrams, by convention, delineated regions (encircled ones in Figures 1A–C) represent sets or collections. (By definition, a set *contains* its members, like a field of sheep contains sheep, whereas a collection *consists* of its members, like a flock of sheep consists of sheep; Moktefi, 2015. Furthermore, any member can appear only once in a set but multiple times in a collection; Bricken, 2019a). Although it is not self-evident that such a delineated region as a circle should represent a set or a collection, thinking of it as a highly simplified drawing of a field or a flock certainly renders it more iconic and intuitive than does a capital letter P or Q.

In Euler diagrams, the positions of the regions relative to one another express the logical interrelationships between sets or collections, and in this way they are also iconic (Moktefi, 2015). For example, by representing the set of ewes inside the set of sheep (rams and ewes), it becomes immediately obvious that all ewes are sheep (Figure 1A); by letting the set of mammals overlap with the set of sea creatures, it becomes immediately obvious that some, but not all, mammals are sea creatures and vice versa (Figure 1B); and, by representing sheep separately from goats, it becomes immediately clear that no sheep is a goat and vice versa (Figure 1C). Venn diagrams (Venn, 1881) resemble Euler diagrams but, instead of manipulating the relative position of delineated regions to express set relationships, these regions are compartmentalized (compare Figures 1D–F). Although Euler and Venn diagrams were developed in, respectively, the 18th and 19th century, they are still popular today. As they become more complex, however, they quickly become hard to read, and the range of logical problems they can handle is also rather limited.

## 2 Existential graphs

Around the beginning of the 20th century, Charles Sanders Peirce, who was not only a founder of the study of signs but also an important contributor to symbolic logic, proposed a more powerful iconic alternative to Euler and Venn diagrams: *existential graphs*. He thought that not his symbolic logic but this iconic alternative “ought to be the logic of the future” (Peirce, 2020, p. 12; see also Peirce, 2021a,b). The iconic logic of existential graphs is particularly interesting because a very similar precursor of these graphs (*entitative graphs*) has been developed into a more general *iconic mathematics* (for a psychologist’s take of iconic mathematics, see Kramer, 2022; for mathematicians’ and computer scientists’ expositions, see Spencer Brown, 1969; Kauffman, 1995; Bricken, 2019a,b, 2021; Kauffman et al., 2023).

Peirce offered three kinds of existential graphs, which he rather unhelpfully—and, as we shall see later, rather tellingly—called “alpha,” “beta,” and “gamma.” I call them instead, in line with their symbolic counterparts, *existential propositional*, *existential predicate*, and *existential higher-order and modal logic*. Peirce did not complete the latter, and I will only discuss the former two (but see Peirce, 2021a and Roberts, 1973; Dau, 2006). Along the way, I will revive some of Peirce’s ideas that he himself deemed impractical but that, thanks to modern technology, no longer are and that—as a surprising byproduct—render his graphs quite decorative; suitable, quite frankly, to hang on a wall as art.

### 2.1 Black-and-white thinking

#### 2.1.1 Sketching out premises

**Figure 2:**
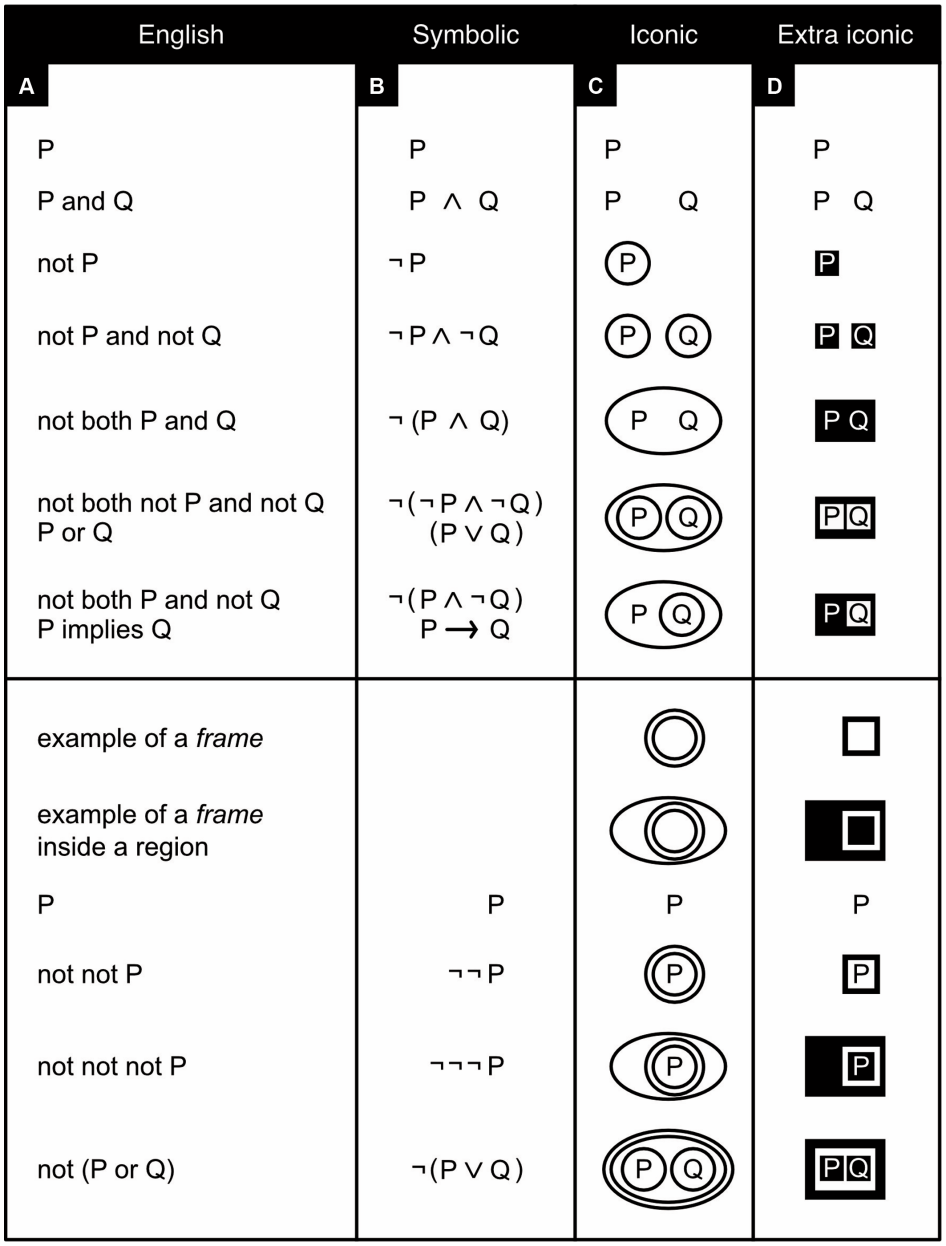
Existential graphs. Several logical relationships are expressed in English **(A)**, symbols **(B)**, traditional existential graphs **(C)**, and black-and-white existential graphs **(D)**. Note that some logical relationships can be expressed in two different ways in both English **(A)** and symbols **(B)** but in just one way in traditional **(C)** or black-and-white **(D)** existential graphs. The bottom half of the figure shows examples of what I call *frames* (which Peirce called “double cuts,” with each individual boundary called a “cut”). Here “or” means “and/or” (inclusive, rather than exclusive, or).

To construct his existential graphs, Peirce drew on a sheet of paper (*sheet of assertion*) regions enclosed by circles, ovals, or other shapes. These regions could be nested within each other but, for the graphs to be considered “well-formed,” they were not allowed to overlap^1^ (Figure 2C). Within any region or subregion one can write *propositions* (statements that are either true or false) or capital letters like P, Q, and R that stand for such propositions. Those written within the most encompassing region (the sheet of assertion itself) mean what they say (P is P); those within its subregions mean the negation of what they say (P becomes “not P”); those within its sub-subregions, the negation of the negation of what they say (P becomes “not not P”), and so on. Such drawings of regions with propositions written in them form the starting points (*premises*) for rigorous logical arguments that are *valid* if they strictly follow a bespoke set of transformation rules and *sound* if, in addition, the premises happen to be true. Sound arguments render conclusions indisputably true that otherwise may at best merely seem to be true.

**Figure 3:**
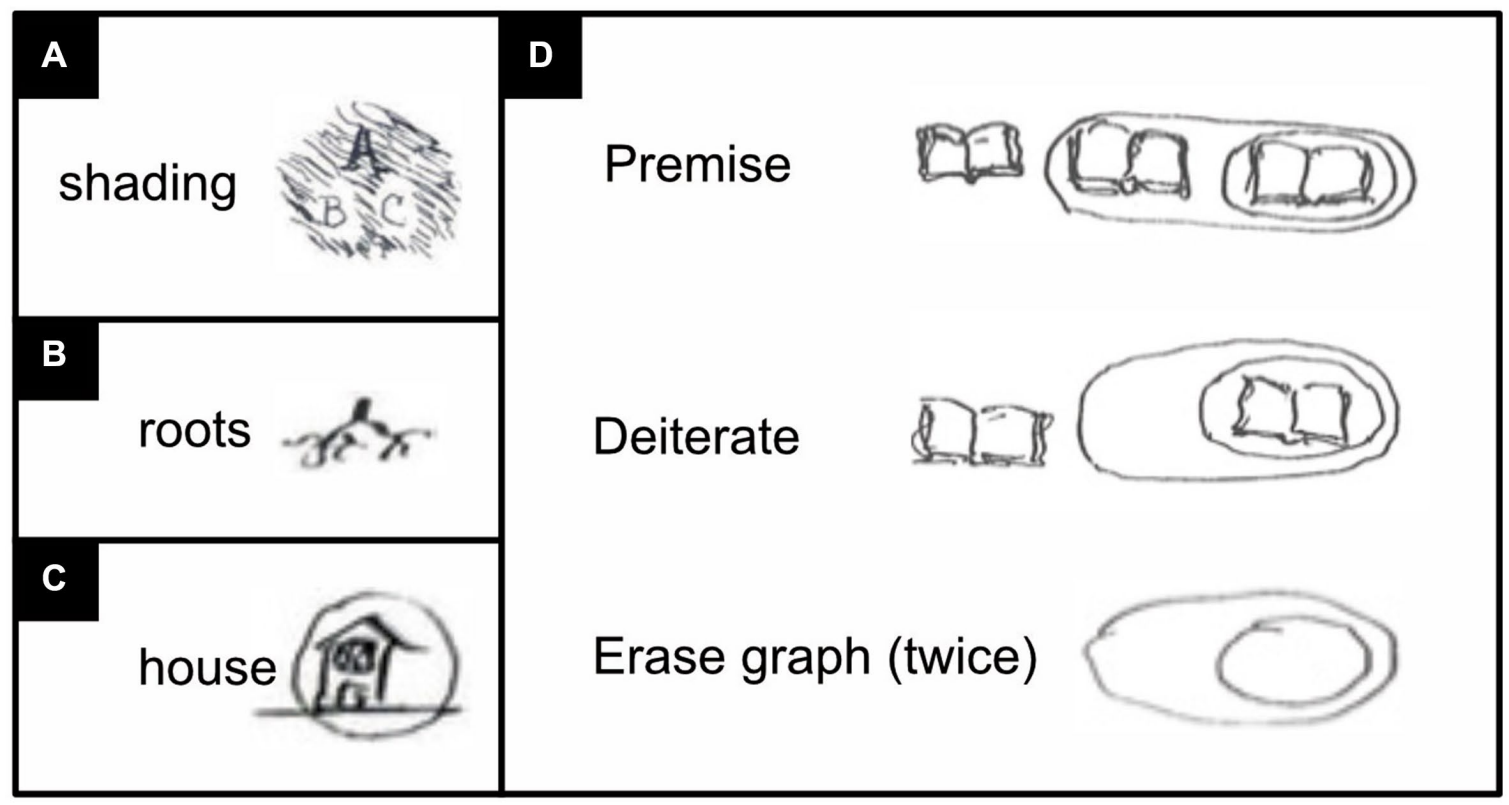
Rare graphs and pictures by Peirce’s own hand (Peirce, 2020, p. 483–484, 571). **(A)** A graph with “not A” expressed with the help of shading. The expression in its entirety reads: “not A or (B or C)” or equivalently: “not (A and both not B and not C)” or equivalently: “A implies (B or C)”. **(B)** A picture of plant roots. **(C)** A picture of a house that has been encircled to express the negation of its meaning. **(D)** A logical argument that, instead of words, uses a picture of an open book—here intended to visualize “tautology”. For the logic of the argument, see the main text below.

In his graphs, Peirce almost always drew only the outlines of regions (Figure 2C); by and large, modern iconic logicians follow him in this practice. Occasionally, however, he shaded regions that negate something (Figure 3A; Peirce, 2020, p. 571). Perhaps because we are diurnal rather than nocturnal, across cultures we tend to associate positive things with the light color of white and negative things with the dark color of black (Jonauskaite et al., 2020). Peirce’s idea to use shading for negation is thus quite intuitive and ergonomic. Still, reminiscent of the fact that the negation of a negation amounts to a positive affirmation, a region’s subregions were left unshaded, sub-subregions were shaded again, sub-sub-subregions unshaded, and so forth.

Unfortunately, Peirce deemed the shading practice “insufferably inconvenient” (Peirce, 2020, p. 571) for the scribe (the person who draws graphs and adds text to them). And although Peirce later did use shading again after all, his followers today still tend not to. With modern software, however, scribing convenience is no longer an issue. To better serve the reader, therefore, it would seem preferable to reinstate Peirce’s discarded idea (Figure 2D), which has the added benefit of rendering nested regions easier to distinguish and to refer to. When shading is not used, regions are now referred as being either oddly or evenly enclosed. Whether a region is surrounded by an odd or even number of boundaries, however, is rarely as obvious as whether this region is shaded or not. Using shading is thus more ergonomic.

**Figure 4:**
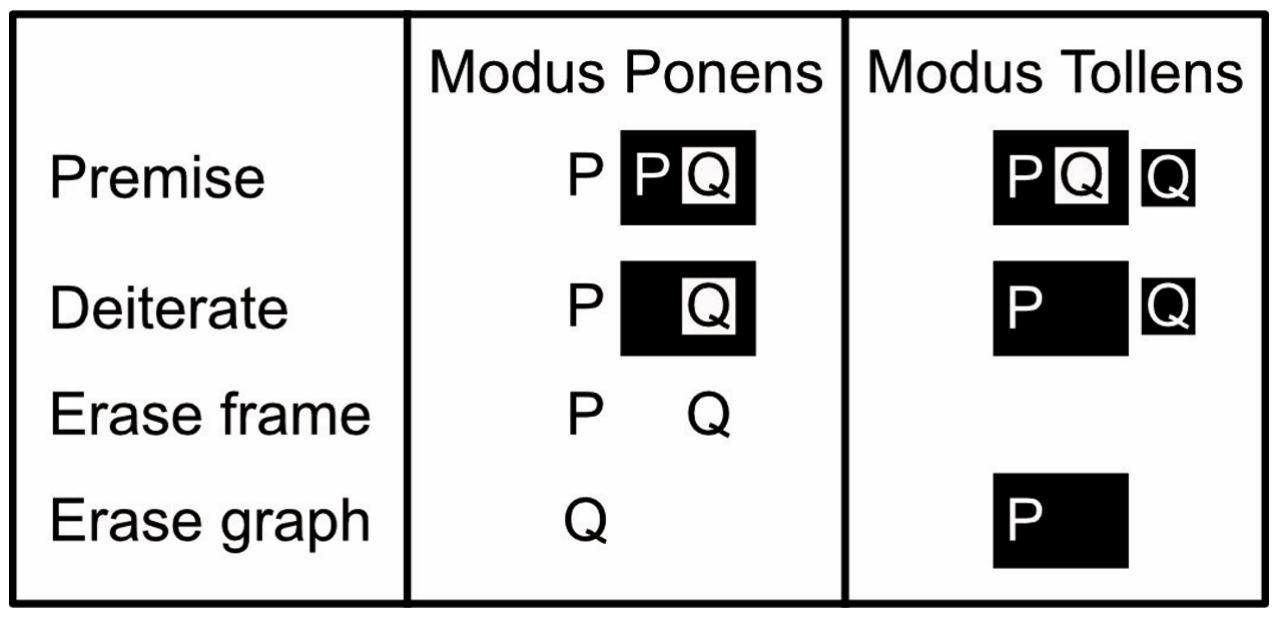
Basic existential propositional logic with letters. *Modus Ponens* argument: given, as premises, that P is true and that “P implies Q” is valid, we can infer that Q is true. Rephrased, the premise states that P is true and that it is not the case that P is false and Q is true. Given that P is true, the Deiterate rule eliminates from further consideration the idea that P could be false (i.e., the white-on-black P is erased). The Erase frame rule then rephrases “not not Q” as just “Q” (i.e., the black frame around the black-on-white Q is erased). And the Erase graph rule finally allows us to conclude that given that P and Q is true, Q on its own is true as well (i.e., P can be erased). *Modus Tollens* argument: given, as premises, that “P implies Q” is valid, but that Q is false, we can infer that P is false. Given that Q is false, the Deiterate graph rule eliminates the idea that Q could be true from further consideration (i.e., the black-on-white Q is erased). The Erase graph rule now allows us to conclude that given that P is false and Q is false, P on its own is false too (the white-on-black Q can be erased). (Here, unlike the Modus Ponens argument, the Modus Tollens one does not need the Erase frame rule).

**Figure 5:**
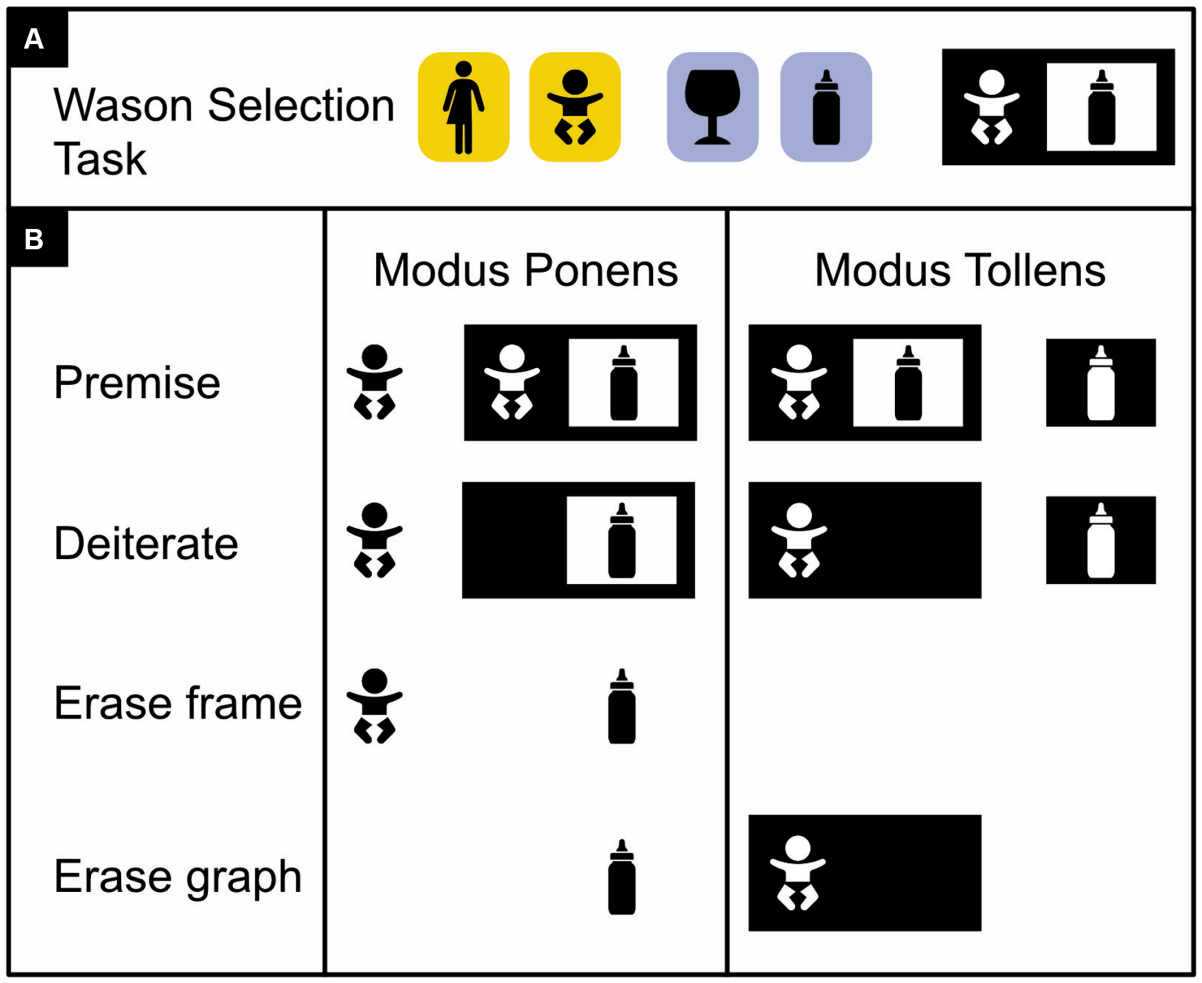
Basic existential propositional logic with icons instead of words or letters. **(A)** Illustration of a *Wason selection task* (Wason, 1968, 2013). Four cards are shown with either an adult or a baby on one side (on yellow) and either a cognac glass or baby formula on the other (on blue). The task is to indicate which of these four cards *must* be turned over to ascertain that a card with a baby on one side depicts baby formula on the other. The correct answer is that the card depicting the baby must be turned over, to check whether—per Modus Ponens—the other side correctly features baby formula, and the card depicting the cognac glass must be turned over, to check whether—per Modus Tollens—the other side correctly depicts an adult and not a baby. **(B)** Modus Ponens and Modus Tollens arguments with concrete icons instead of abstract words or letters. (Icons from thenounproject.com; for acknowledgements, see Section 6).

Some of Peirce’s work has been lost, but in what has survived he nearly always expresses propositions with the help of words or letters like P and Q (Figures 2C, 3A). In exceedingly rare snippets of text, however, Peirce did not use such abstract tokens but instead tiny little pictures (Figures 3B–D; Peirce, 2020, p. 483–484). Most likely he deemed drawing these pictures “insufferably inconvenient” for scribes too. Yet pictures are literally easier to picture, and also easier to remember, than are abstract words or letters (Kramer, 2023). Using pictures is thus more ergonomic. Modern technology can also make scribing pictures much less trouble than before. It would thus seem preferable to reinstate this idea as well (compare Figures 4, 5). Wherever this seems helpful, I represent propositions and concepts not with abstract tokens but with black-on-white icons and their negation with white-on-black ones (see also Kramer, 2023).

#### 2.1.2 Drawing conclusions

**Figure 6:**
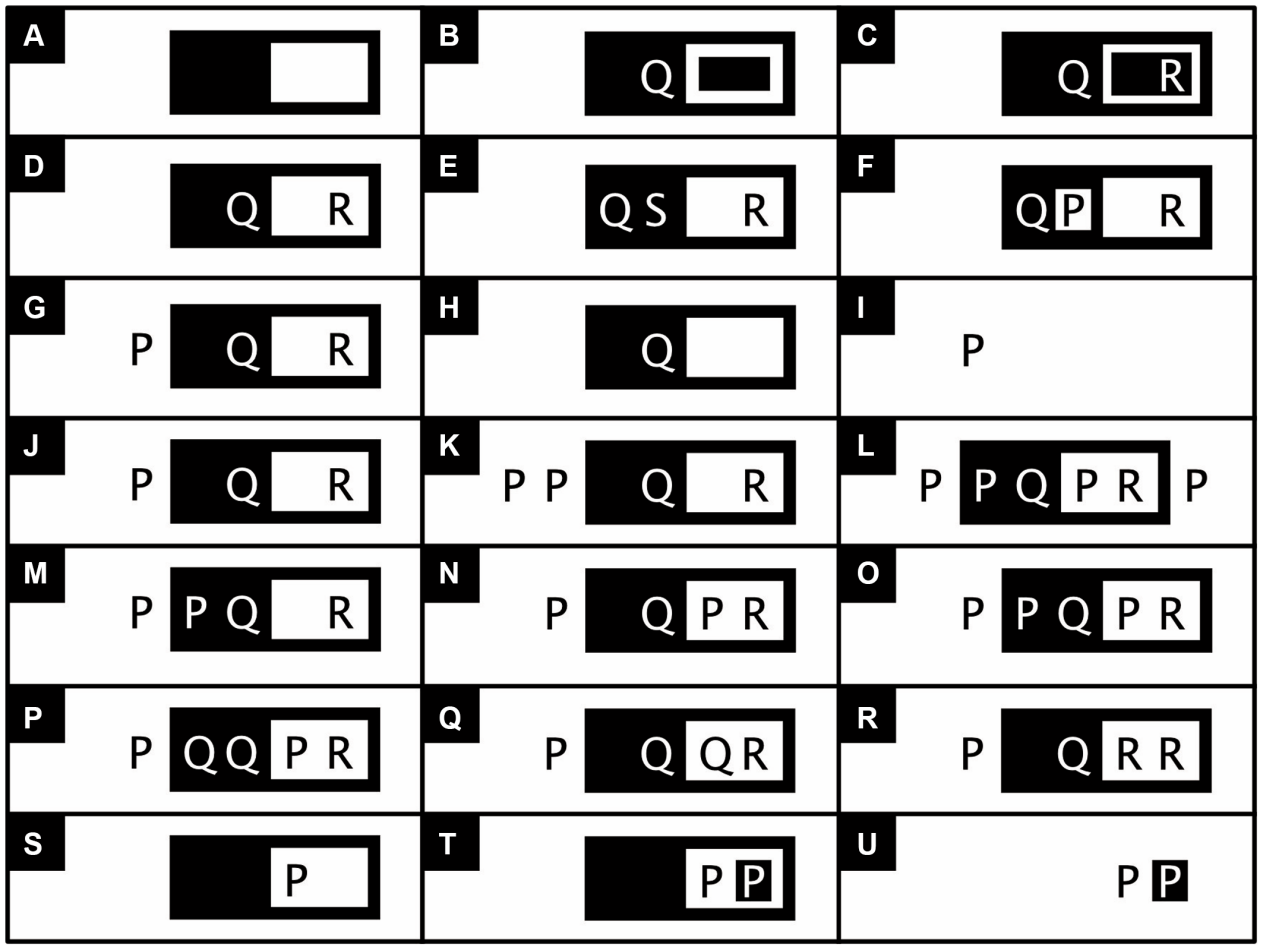
Example applications of the inference rules of existential propositional logic (adapted, except for A–C, and F, from Roberts, 1973, p. 42 and 43, in turn adapted from similar examples by Peirce, 2020, #93981). The *Insert/erase frame* rule permits one to draw a frame on a blank sheet of assertion **(A)** or any other region **(B)** or **(C)** or to erase such a frame. The *Insert graph* rule permits one to transform **(D)** into **(E)** or **(F)**, and the *Erase graph* rule to transform **(G)** into **(H)** or **(I)**. The *Iterate graph* rule permits one to transform **(J)** into any of the graphs shown in **(K)** through **(P)**, and the *Deiterate graph* rule allows one to do the converse. Not permitted is the transformation of **(S)** into **(T)**. This transformation copies a subgraph of **(S)**, consisting of the innermost region of **(S)** and its content (the graph P), onto the subgraph itself (in reversed colors and redimensioned). Such a transformation would lead to a contradiction, most clearly seen when the erase frame rule is used to transform **(T)** into **(U)**.

**Figure 7:**
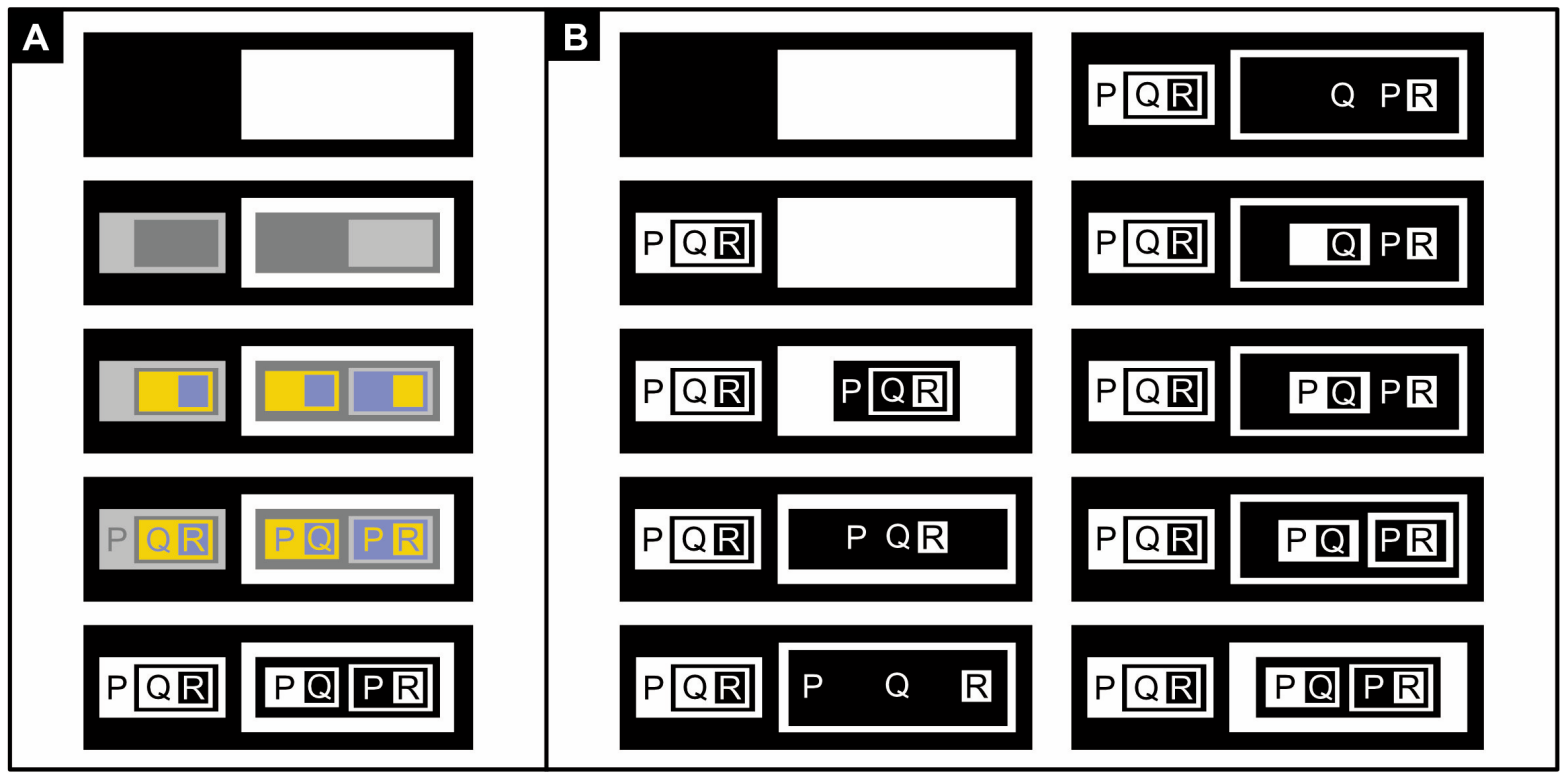
Relatively complex existential propositional logic (adapted from Roberts, 1973, p. 46). **(A)** The iconic form of the *Distributive Law of Implication*: [P → (Q → R)] → [(P → Q) → (P → R)]. In symbolic logic, the distributive law has a hierarchical structure, typically elucidated with brackets within brackets. In iconic logic, the nested brackets are replaced with nested regions. To clarify the hierarchical structure of these nested regions, I first show those involved at the highest level in black and white (line 1), those at the intermediate level in dark and light gray (line 2), and those at the lowest level in blue and yellow (line 3). Subregions within light regions (the white, light gray, and yellow regions) are represented in the color opposite to those within dark regions (the black, dark gray, and blue regions). Likewise, propositions are also shown in the opposite color as the regions in which they are placed (line 4). The final representation of the distributive law in existential propositional logic is obtained by painting all dark regions black and all light regions white (line 5). **(B)** Proof of the distributive law in ten steps, from top to bottom and left to right, using: (1) the Insert frame rule, (2) the Insert graph rule, (3) the Iterate graph rule (and reversing fore-and background colors), (4) the Erase frame rule (and redimensioning a bit), (5) some reorganization, (6) some more reorganization, (7) the Insert frame rule, (8) the Iterate rule, (9) the Insert frame rule, and (10) some final adjustment.

To draw valid conclusions from premises expressed with existential graphs, inference rules need to be followed. These rules allow one to transform a graph that expresses the premises of an argument into one that validly expresses its conclusion (Figures 4-7). To keep these rules simple, for existential propositional logic, I define as a *graph* anything consisting of one or more regions and/or propositions, and as a *frame* any region without content that completely encloses another region that itself may, or may not, have any content (Figures 2C,D, bottom half). Originally, the rules refer to regions as either oddly or evenly enclosed; reviving Peirce’s use of shading, my reformulation of them refers to these same regions as, respectively, either black (shaded) or white (unshaded). Peirce’s *existential propositional logic* has proven to be both *sound*, which here means that it cannot lead to contradictions, and *complete*, which here means that all true assertions can be derived from an empty sheet of assertion by no more than the application of the inference rules (Dau, 2006; Caterina et al., 2022; see also Peirce, 2020). These inference rules permit nothing except the following (reformulated from Dau, 2006):

1. *Insert/erase frame*: Draw or erase a frame inside any region.
2. *Insert/erase graph*: Draw any graph inside a black region or erase any inside a white region.
3. *Iterate/deiterate graph*: Draw a copy of a subgraph inside an encompassing graph in either the same region or one further inward, or erase such a copy (for visibility, draw any copy in the color opposite to that of its local background). Do not copy any part of the subgraph onto this subgraph itself.

After impressionism, expressionism, abstract art, computer art, and numerous other artistic genres, some have argued that we have seen it all, and that art has nothing new to offer anymore. Yet it seems unprecedented that logical inferences can become as decorative as paintings. Some logical diagrams do contain illustrations alongside ordinary text (Berger, 2017; Even-Ezra, 2021) and many diagrams have some artistic aspects (Elkins, 1995; Stjernfelt, 2017). Yet, Figure 7B, say, could be mistaken for an example of lettrism, in which letters are used purely for esthetic reasons; if its letters were replaced by icons, the figure would be even less recognizable as an inference or practically useful diagram. Only half in jest, therefore, I am tempted to call such iconic proofs *inferential art* and to entitle Figure 7B “composition of dark versus light”—or more provocatively, but meaningfully—“black and white thinking.”

#### 2.1.3 Drawing logic into algebra

**Figure 8:**
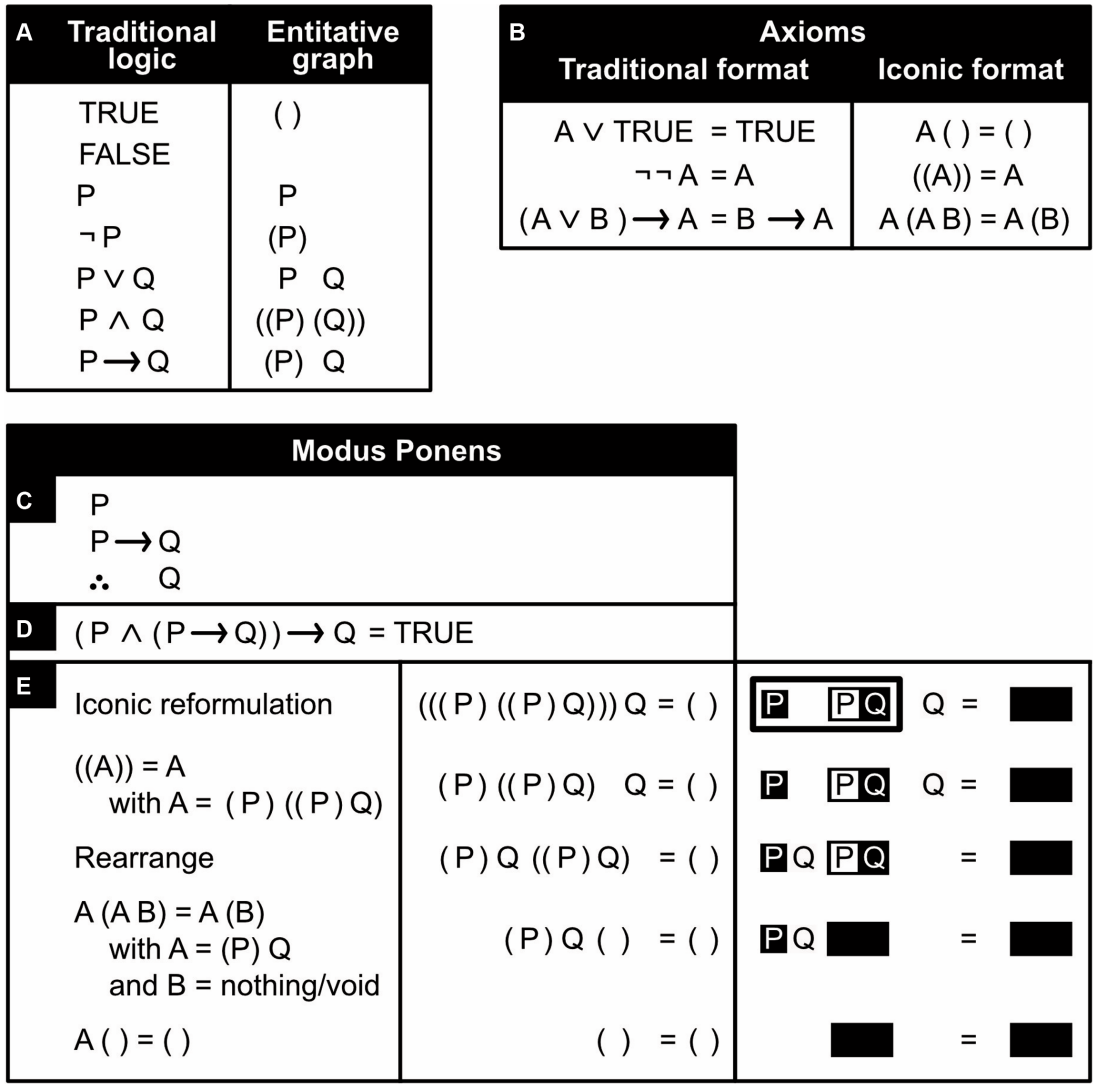
An iconic alternative to Boolean algebra. **(A)** Traditional, symbolic logical notation (left) and its entitative-graph equivalent (right). In the latter, truth is replaced by the confirmation of the existence of something, or more precisely, by the denial of the existence of nothing (i.e., by an empty region enclosed by a pair of brackets) and falsehood by literally nothing. A proposition is expressed with a capital letter (e.g., P) and its negation by enclosing this letter inside a region (inside a pair of brackets). **(B)** Basic equations (*axioms*) assumed to be valid. (Note how much more concise and intuitive they are, compared to Peirce’s inference rules.) **(C)** A Modus Ponent argument expressed in traditional, symbolic logic (the triangle of three dots means “therefore”). **(D)** The same argument expressed as an equation. **(E)** Proof of the Modus Ponent argument: first line: the argument reformulated as an entitative graph, using either brackets (middle column) or the black-and-white notation of previous figures (right column). Second line: using the axiom *((A))* = *A* to simplify the equation. Third line: rearranging terms. Fourth line: using the axiom *A (A B)* = *A (B)* to simplify the equation further. Fifth line: using the axiom *A ( ) = ( )* to arrive at the obvious tautology that proves the argument.

Besides uniting it with art, one can also unite logic with math. That is, one can reformulate logical derivations as equations and turn logic into algebra (Dunn and Hardegree, 2001; Boole, 2009). This *Boolean algebra* is popular in electrical engineering and its *fuzzy logic* extension in artificial intelligence. An iconic alternative to it has been developed that is based on entitative graphs (Figure 8; Spencer Brown, 1969; Bricken, 2023). Entitative graphs can be converted into existential graphs and vice versa but in the former, unlike the latter, *P Q* does not express *P and Q* but *P or Q* (with “or” being inclusive rather than exclusive, meaning “and/or”). With a pair of brackets delineating a region, it follows that (*(P) (Q)*) expresses *not (not P or not Q)*, which equals *P and Q*. *P implies Q* is expressed as *(P) Q* or, in other words, as *not P or Q*. The desired goal of a logical derivation is that it ends with a conclusion, the desired goal of its algebraic equivalent that it ends with a tautology—an equation that, under all circumstances, is trivially and undeniably correct.

### 2.2 Adding color to our thoughts

**Figure 9:**
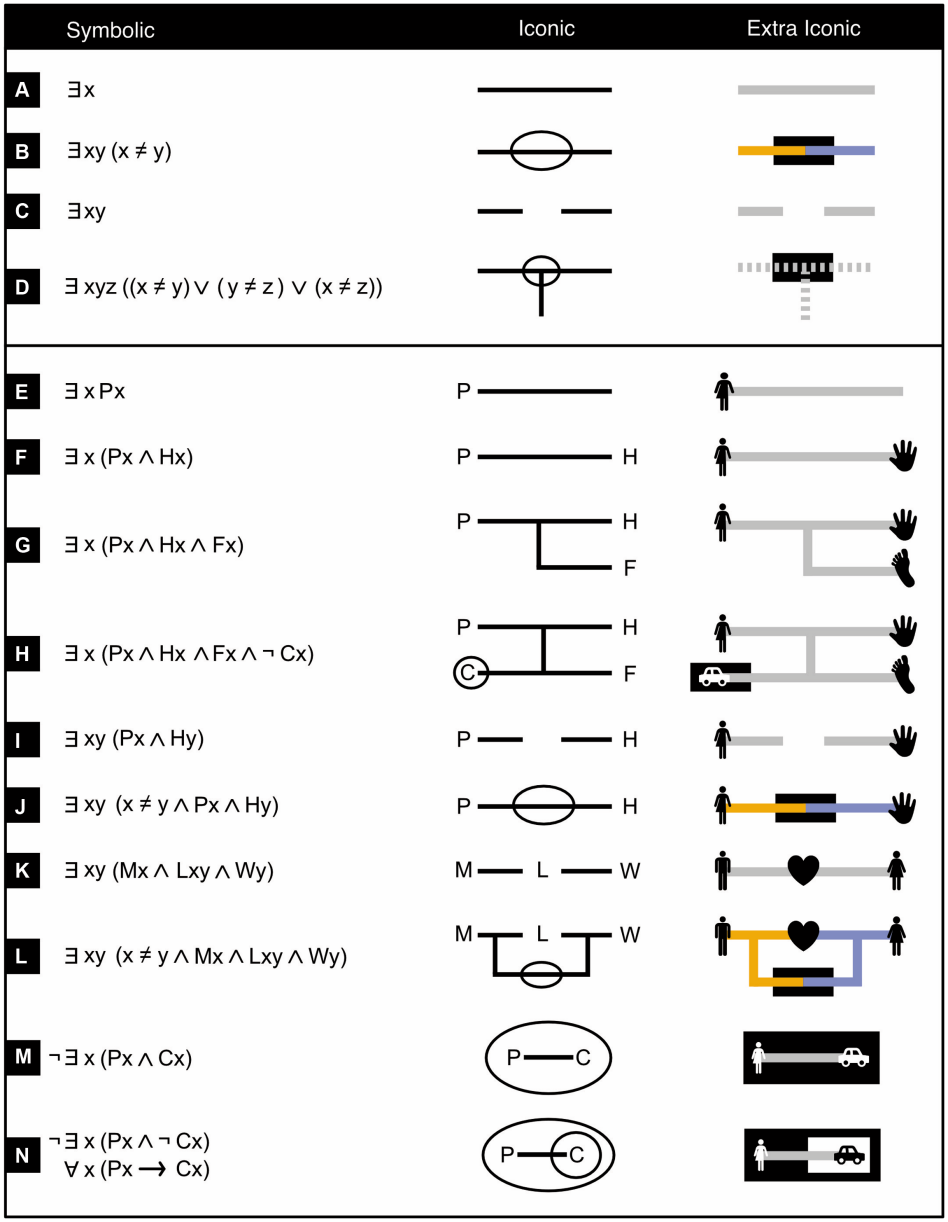
Ligatures in existential graphs. Symbolic logical expressions (left column) and equivalent existential graphs (middle and right columns) that feature ligatures (continuous, dashed, or dotted lines or networks of lines) and predicates (capital letters in the middle column, icons in the right one). ∃*x*, ∀*x*, ¬, ∧, ∨, → respectively mean “there exists at least one *x*,” “all or every *x*,” “not,” “and,” “or,” “implies”. P means “is a person,” H “has hands,” F “has feet,” C “has a car,” M “is a man,” L “loves,” W “is a woman”. Note, in line N, that some existential graphs can be translated into symbolic logic in two or more mutually equivalent ways. (Icons from thenounproject.com; for acknowledgements, see Section 6).

In order to reason about not assertions but individual persons, objects, or other items that are mentioned within such assertions, Peirce enriched his existential graphs with networks (*ligatures*) consisting of one or more line segments (*lines of identity*) that bind (*ligare*, in Latin) items to their properties or relationships with other items (Figure 9, middle column). To one’s liking, lines of identity can be drawn straight, curved, or even meandering. Properties and relationships are described in attached labels (*predicates*). The ligatures with their predicates effectively turn existential propositional logic into existential predicate logic. To help the reader recall what the predicate labels stand for, I once again use icons instead of words or letters (Figure 9, right column).

#### 2.2.1 Making sense logically but not psychologically

Although existential graphs are more iconic, and thus likely easier to picture and more intuitive than traditional symbolic predicate logic, they are not yet as intuitive as they could be. What is intuitive is that a single line of identity may represent a single item (Figure 9A, middle). Its equivalent in symbolic predicate logic uses, instead of this single line, a single variable like *x* or *y* (Figure 9A, left). Yet if a line of identity completely passes through a subregion (which expresses negation), then even though it is just one line, it is nonetheless supposed to represent two distinct items (Figure 9B, middle). This is quite counterintuitive, because as any introduction to perceptual organization attests, humans strongly tend to perceive a uniformly connected, uniformly colored, and smoothly continuing line as forming one single whole and not two different ones (Kimchi et al., 2003; Wagemans, 2015). Indeed, in symbolic predicate logic (Figure 9B, left), instead of one single line, two distinct variables are used (say, *x* and *y*) and it is then stated that these two variables are not identical (*x* ≠ *y*).

Conversely, intuitively enough, two unconnected lines of identity may represent two different items (Figure 9C, middle). In symbolic predicate logic, instead of two unconnected lines, two different variables are used, like *x* and *y* (Figure 9C, left). Yet even though the two lines of identity are unconnected, they may nonetheless potentially represent just one item. This can occur, for example, when it is not known that the two items represented by these two lines are in fact one and the same (compare Figures 9B,C, middle; only Figure 9B unambiguously represents two items and never just one). Likewise, in symbolic logic, when two variables are used (say, *x* and *y*), then as long as it is not known that these two variables are unequal (*x* ≠ *y*), they may nonetheless potentially represent one and the same item as well—that is, it is possible that *x* = *y* (compare Figures 9B,C, left).

#### 2.2.2 Making sense logically and psychologically

Although Peirce’s system works well from a logical point of view, as just laid out, it does not do so quite as well from a psychological one. For future investigation, I therefore propose a superficial change of the system that leaves its logical features intact but that, within the limits of this constraint, may improve its ergonomic features. More specifically, I propose that related items are interconnected with solid, identically colored, lines of identity (Figure 9, right column, panels A, E, F, G, H, K, M, N), that unrelated items are interconnected with solid, distinctly colored, lines of identity (Figure 9, right column, panels B, J, L) and that items that may, or may not, be related to each other are either interconnected with dashed lines of identity (Figure 9, right column, panels D) or left unconnected (Figure 9, right column, panels C, I).

Consider three particularly instructive examples. First, in Figure 9D, not all lines of identity represent the same item, but because it is not clear which ones do and which do not, they are interconnected with dashed lines. Second, in Figure 9K, there are two lines of identity that represent, respectively, a man and a woman. The two individuals could be distinct, but from a purely logical rather than a biological point of view, they could just as well be one and the same person. So, the two lines of identity are not connected to each other. Third, Figure 9L is similar to Figure 9K, but now the man and the woman are explicitly stated to be different. In this case, therefore, the two lines of identity are solid, interconnected in a subregion (here black), and colored differently.

The rules of inference (discussed a little later) consider the connections between lines of identity but not their appearances. By keeping it that way, the system thus continuous to work as before, but now—thanks to its new appearance—it ought to be easier to read.

#### 2.2.3 Linking individual existence to universal truth

**Figure 10:**
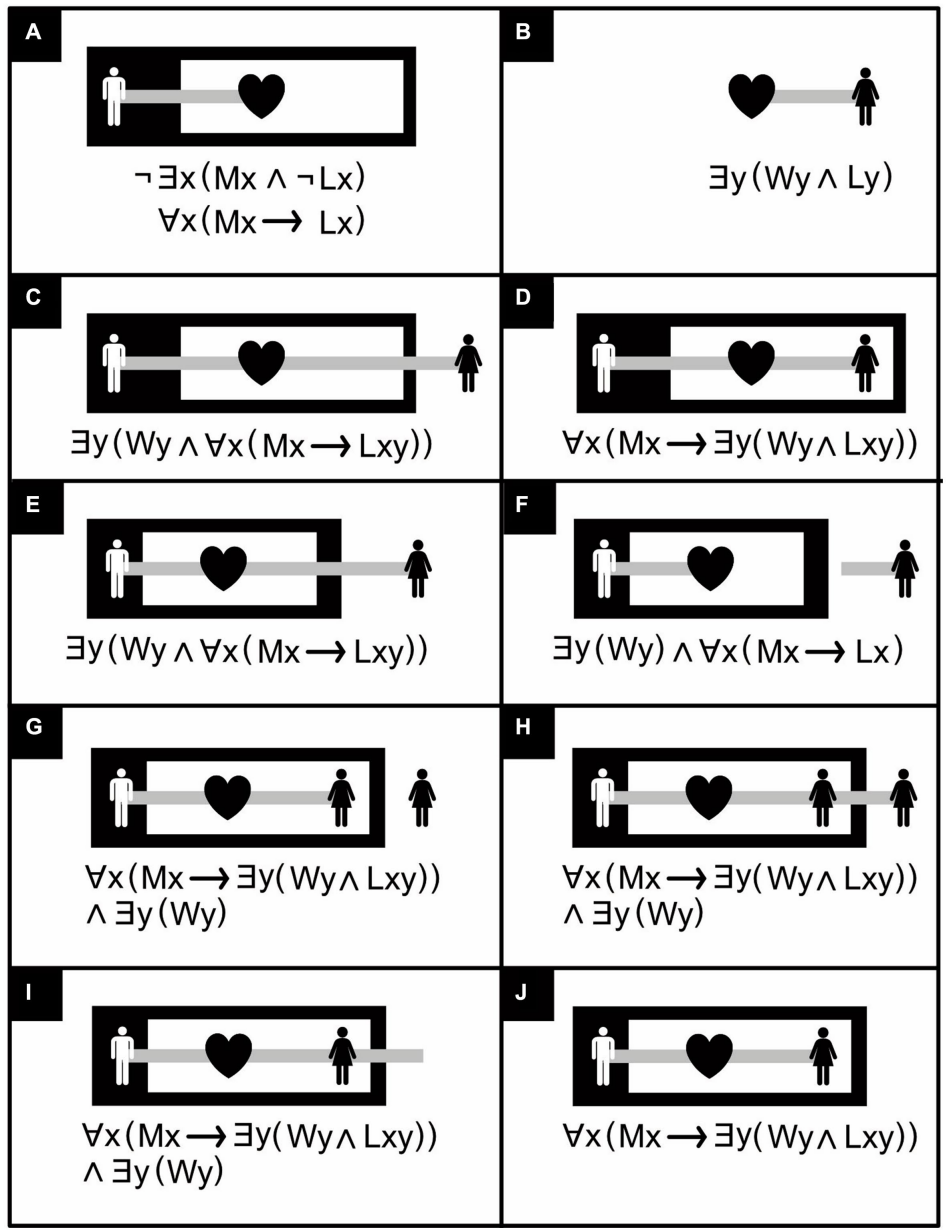
Individual existence and universal truth in existential graphs with ligatures. ∃*x* means “there exists at least one *x*,” ∀*x* “all or every *x*,” ¬ “not,” ∧ “and,” → “implies,” *Mx* “*x* is a man,” *Wy* “*y* is a woman,” *Lx* “is in love,” *Ly* “is loved”. **(A)** “No man is not in love,” or equivalently, “all men are in love”. The two equivalent assertions share the same notation in existential predicate logic but two different ones in their symbolic counterpart. **(B)** “Some woman is loved”. **(C)** “There is some woman, and all men love her”. **(D)** “All men love some woman” [**(C)** asserts the existence of something; **(D)** does not]. **(E)** There is some woman, and all men love her [copy of **(C)**]. **(F)** There is some woman, and all men are in love. **(G)** “There is some woman, and all men love her” (alternative to **(C)**, assuming the woman-icons are tokens, rather than types of tokens, and refer to the same woman and not to potentially different ones). **(H)** “There is some woman, and all men love her” [another alternative to **(C)**]. **(I)** “There is some woman, and all men love her” [yet another alternative to **(C)**]. **(J)** “All men love some woman” [copy of **(D)**]. (Icons from thenounproject.com; for acknowledgements, see Section 6).

Regardless of whether it is attached to a predicate, the outermost part of a ligature indicates whether the item represented by this ligature exists or not (Figure 10). If the outermost part is located on the white sheet of assertion, the item’s existence is asserted; if located in a black subregion, it is denied; if located in a white sub-subregion, its denial is denied, and so on. A combination of denial (negation) and existential quantification (assertion of an item’s existence) can amount to a universal quantification (assertion of a universal truth). Unlike in ordinary symbolic logic, in existential predicate logic there is no separate notation for universal quantification, and the same existential graph can thus sometimes have more than one symbolic interpretation (Figure 10A). In fact, keeping things as simple as possible, existential graphs often have the same representation for various syntactically different, but logically equivalent, symbolic expressions. For example, in existential graphs, the following three symbolic expressions are all represented in exactly the same way: ∃x; ∃x∃y (*x* = *y*); and ∃*x*∃*y*∃*z* (*x* = *y* ∧ *y* = *z* ∧ *x* = *z*) (Bellucci and Pietarinen, 2021).

#### 2.2.4 Drawing conclusions

**Figure 11:**
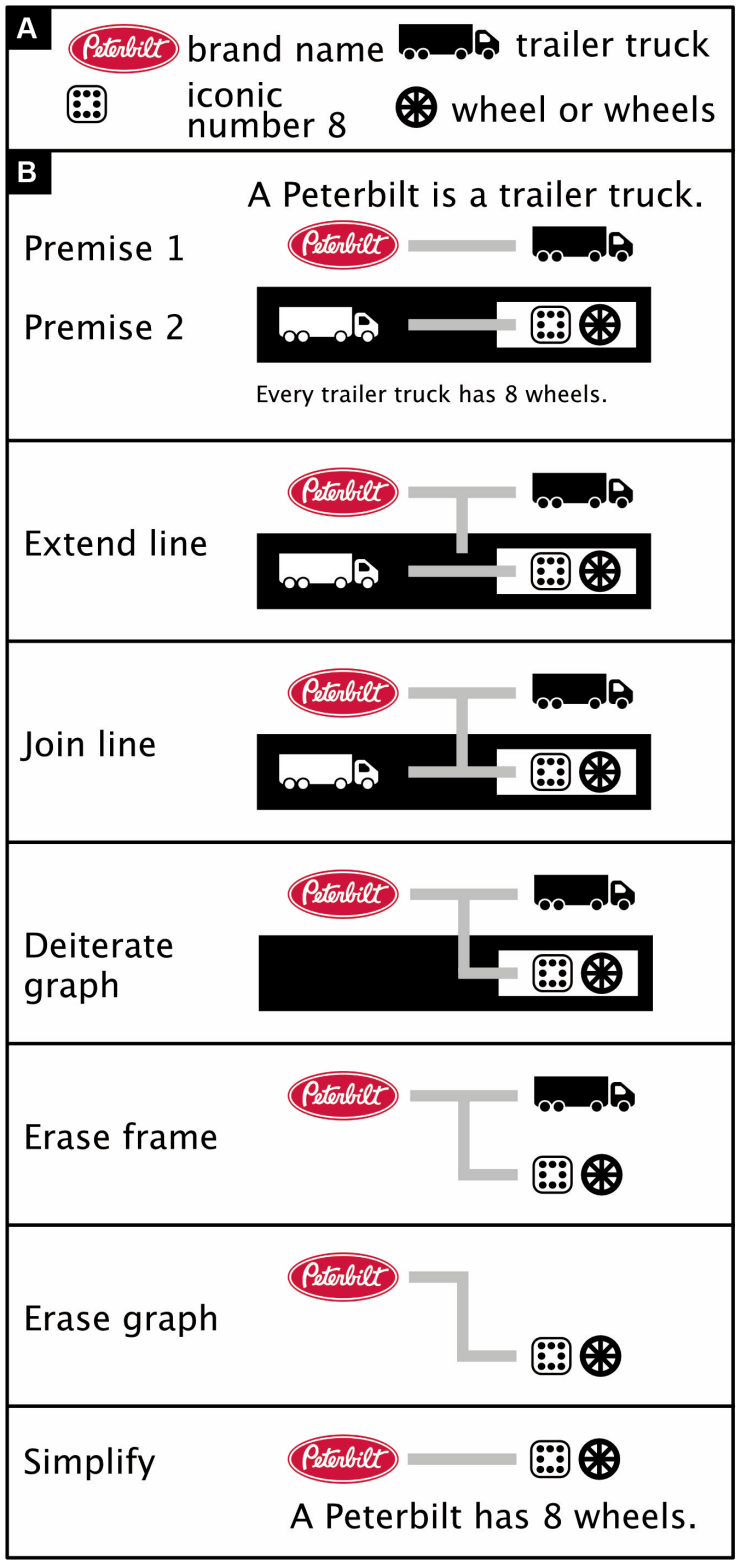
Basic existential predicate logic (adapted from Dau, 2006, pp. 13–14, in turn adapted from similar examples by Peirce, 2020, #93981). **(A)** Icon meanings. **(B)** An iconic proof of a syllogism. Premise 1: A Peterbilt is a trailer truck. Premise 2: Every trailer truck has eight wheels. Here, the Extend line rule expresses the fact that the item has the property of being both a Peterbilt and a trailer truck but does not have some unspecified property (i.e., the item’s ligature is extended into a black region but not connected to anything). The Join line rule expresses the fact that the item is either not a trailer truck or has 8 wheels. Given that the item is a trailer truck, the Deiterate graph rule eliminates from further consideration the idea that it would not be (i.e., the white-on-black trailer truck is erased). The Erase frame rule rephrases “not not having 8 wheels” as just “having 8 wheels” (i.e., the black frame around the black-on-white “eight wheels” is erased). The Erase graph rule now allows us to conclude that, given that the item is a trailer truck and has 8 wheels, the item has 8 wheels (i.e., the black-on-white trailer truck can be erased). (For the logic behind iconic numbers, here the number 8, see Kramer, 2022. Truck icon from thenounproject.com; for acknowledgements, see Section 6).

With minor adjustments, the rules of inference for existential predicate logic are identical to those for existential propositional logic but two rules are added that specifically regulate line-of-identity transformations. For existential propositional logic, I defined as a *graph* anything consisting of one or more regions and/or propositions. For existential predicate logic, I define it as anything consisting of one or more regions and/or lines of identity and/or predicates. Moreover, whereas in existential propositional logic a frame may not have any content, in existential predicate logic an exception is made for lines of identity that completely traverse the frame and do not terminate, or form junctions, within its boundaries (Dau, 2006). In Figure 11B, for example, the graph that follows the label “Deiterate graph” contains a line of identity that goes from a white region to another white region and in the process completely traverses a black region. This black region has no other content and is thus considered a frame. Like his existential propositional logic, Peirce’s *existential predicate logic* has also been proven to be both *sound* and *complete* (Dau, 2006; Gangle and Caterina, 2015; see also Peirce, 2020). Its inference rules permit nothing except the following (reformulated from Dau, 2006):

1. *Insert/erase frame*: Draw or erase a frame inside any region.
2. *Insert/erase graph*: Draw any graph inside a black region or erase any inside a white region.
3. *Iterate/deiterate graph*: Draw a copy of a subgraph inside an encompassing graph in either the same region or one further inward, or erase such a copy (for visibility, draw any copy in the color opposite to that of its local background). Do not copy any part of the subgraph onto this subgraph itself.
4. *Extend/retract line*: Extend a line of identity (or branch of it) inward or retract it outward.
5. *Join/disjoin line*: Join lines of identity in a black region or disjoin them in a white one. Join or disjoin copies of the same graph.

The Extend/retract line rule permits replacing some assertions with similar, weaker ones but not the converse. For example, the Retract line rule permits replacing the assertion “there is some woman, and all men love her” (Figure 10E) with the weaker one “there is some woman, and all men are in love” (Figure 10F).

The Join line rule permits replacing an assertion about things that are *potentially* distinct (e.g., the man and the woman that are unconnected in the white region of Figure 9K) with a similar assertion in which these things that are *definitely* distinct (e.g., the man and the woman that are connected within a black region in Figure 9L). The converse is forbidden. For example, one may not join the line of identity attached to the man and the line of identity attached to the woman in the white region in Figure 9K or disjoin them in the black region in Figure 9L. Additionally, the Join line rule permits replacing Figure 10G with Figure 10H or vice versa.

Both Peirce (2020) himself and Dau (2006) have offered various versions of these inference rules that differ in their details. Roberts (1973) presents a version of Peirce’s rules with an extra constraint on one of them—obtained from Peirce’s writing elsewhere—to stop it from being too permissive. Shin (2002) presents a version that is interestingly permissive in some respects but too permissive (Dau, 2006) in others. Using graph theory, Dau offers a mathematical reformulation of his rules, but it may be overly restrictive. For example, both Dau und Shin have a version of the Deiterate graph rule that permits replacing Figure 10H with Figure 10I, but unlike Dau, Shin not only permits retracting a loose end of a line of identity outward into any region (Retract line rule) but also inward into a white one (as in the transformation of Figure 10I into Figure 10J). Indeed, the assertion that all men love some woman (Figure 10J) is similar but weaker than the assertion that there is some woman who is loved by all men (Figure 10I). One would think, therefore, that replacing the latter (Figure 10I) by the former (Figure 10J) should be permitted. Indeed, Peirce (2020, p. 309) does permit this, but it is not obvious how Dau’s rules would do so.

**Figure 12:**
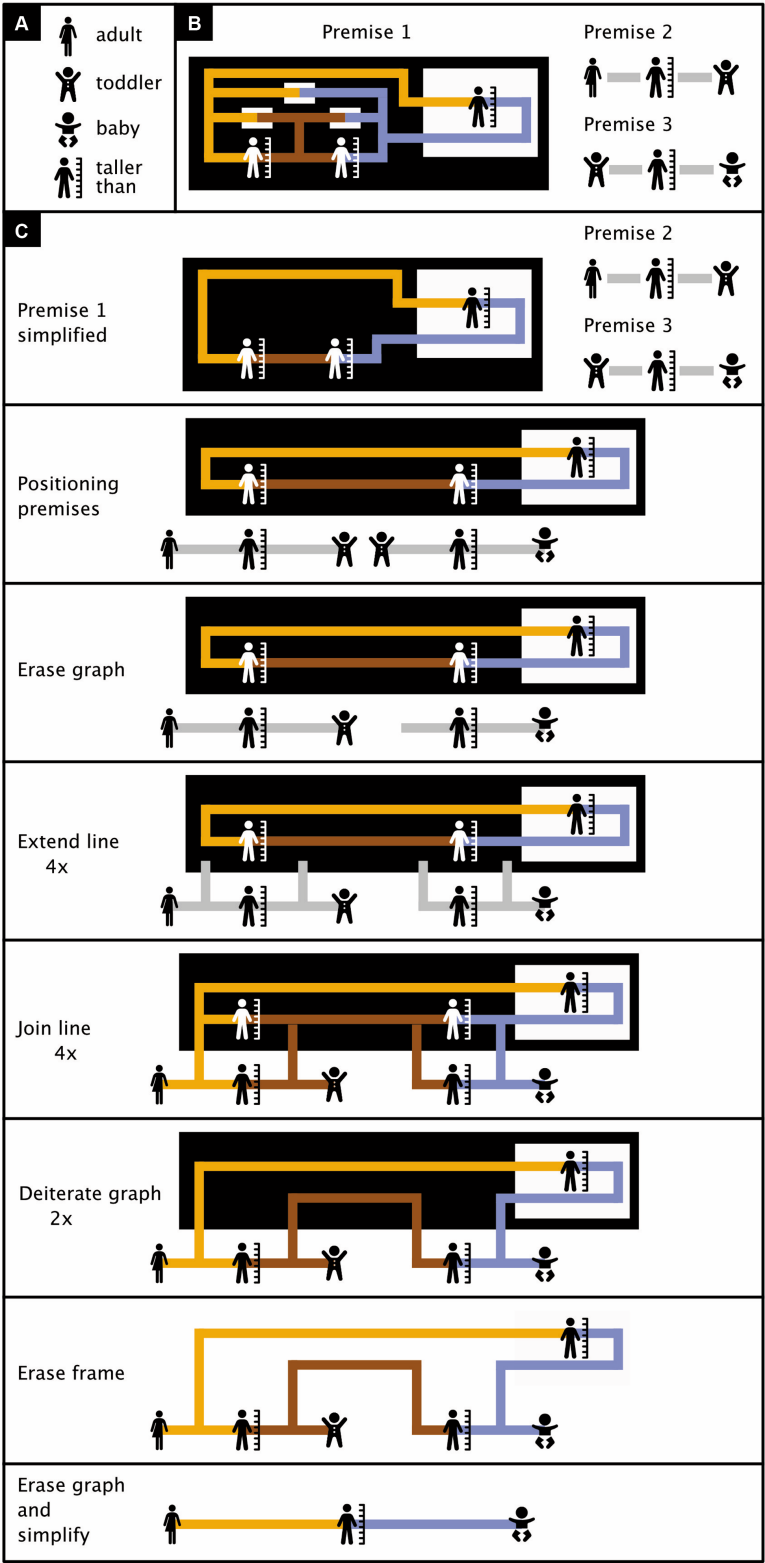
Relatively complex existential predicate logic. **(A)** Icon meanings. **(B)** Premises (see main text for details). **(C)** Proof in seven steps that the adult pictured in Premise 2 must be taller than the baby pictured in Premise 3. (Icons from thenounproject.com; for acknowledgements, see Section 6).

Besides the fact that the rules of existential predicate logic are not fully crystalized out yet, at least one minor extension of the notation is also needed to allow the system to express all of “first order” predicate logic (Bellucci et al., 2020). By and large, however, existential graphs seem a promising alternative to traditional propositional and predicate logic [for more elaborate examples than those in Figure 10, see Figure 11, Figure 12; for the translation of existential logic to symbolic logic, see (Dau, 2006)]. An iconic alternative to Boolean algebra that can handle predicate logic may be even more promising and is in preparation (Bricken, personal communication, 12 September 2023).

Compared to Figure 11, Figure 12 shows a relatively more complex proof. Figure 12A shows the meanings of the icons used in the proof, Figure 12B the three premises that are the starting points of the logical argument, and Figure 12C the proof itself. Premise 1 contains, among other things, three (white) subregions without content that are traversed by a line of identity, and in which this line thus changes appearance. These three subregions play no role of importance in the proof, and for simplicity, they are eliminated in Figure 12C. After this, Premise 1 features a black region with content and a single white subregion with content. This suggests we are dealing with an implication (compare with Figure 9K). Premise 1 states that tallness is transitive—that is, that if Item 1 (yellow ligature) is taller than Item 2 (brown ligature), and if Item 2 (brown ligature) is taller than Item 3 (blue ligature), then Item 1 (yellow ligature) is also taller than Item 3 (blue ligature). Premise 2 states that some adult happens to be taller than some toddler. Premise 3 states that the toddler of Premise 2 happens to be taller than some baby. The task, now, is to prove that the adult mentioned in Premise 2 must be taller than the baby mentioned in Premise 3. The proof consists of the following seven steps:

1. Positioning premises: Purely for convenience, Premises 2 and 3 are placed below Premise 1 and the graph of Premise 1 is elongated and streamlined.
2. Erase graph rule: Of the two toddler icons, one is eliminated.
3. Extend line rule (applied four times): Four lines are extended inward from a white region (the sheet of assertion) into a black one.
4. Join line rule (applied four times): The four lines mentioned in Step 3 are joined with existing lines of identity in the black region (and all joined lines are given the same color).
5. Deiterate graph rule (applied twice): There are two black-on-white “taller than” icons on the sheet of assertion and two oppositely colored copies of them further inward. The inward ones are erased.
6. Erase frame rule: The black region has become a frame and can therefore now be erased as well.
7. Erase graph rule (applied three times) plus simplification: The icon of a toddler and two “taller than” icons are erased, the brown line of identity is erased, and the result is simplified to improve readability. This completes the proof. Premises 1, 2, and 3 together allowed us to conclude, in seven steps, that the adult pictured in Premise 2 is taller than the baby pictured in Premise 3.

## 3 Concept diagrams

Experiments that investigate which systems of logic are best tailored to the human mind are rare and, to my knowledge, none have investigated existential or entitative graphs. Concept diagrams are particularly interesting because they are quite powerful and their user-friendliness has been investigated experimentally. These behavioral studies have been conducted by logicians, mathematicians, and information technology experts—prominent among them John Howse and colleagues (Veres and Mansson, 2004; Shimojima and Katagiri, 2013; Blake et al., 2014; Alharbi et al., 2017; Shams et al., 2018; Stapleton et al., 2020). Here, reviewing (Section 3.1) and then critiquing (Section 3.2) a recent example of such studies (McGrath et al., 2022), I argue that, without doing the logician’s work, behavioral scientists could have made—and still can make—an important contribution to such research.

### 3.1 Using color to better identify sets and relationships

**Figure 13:**
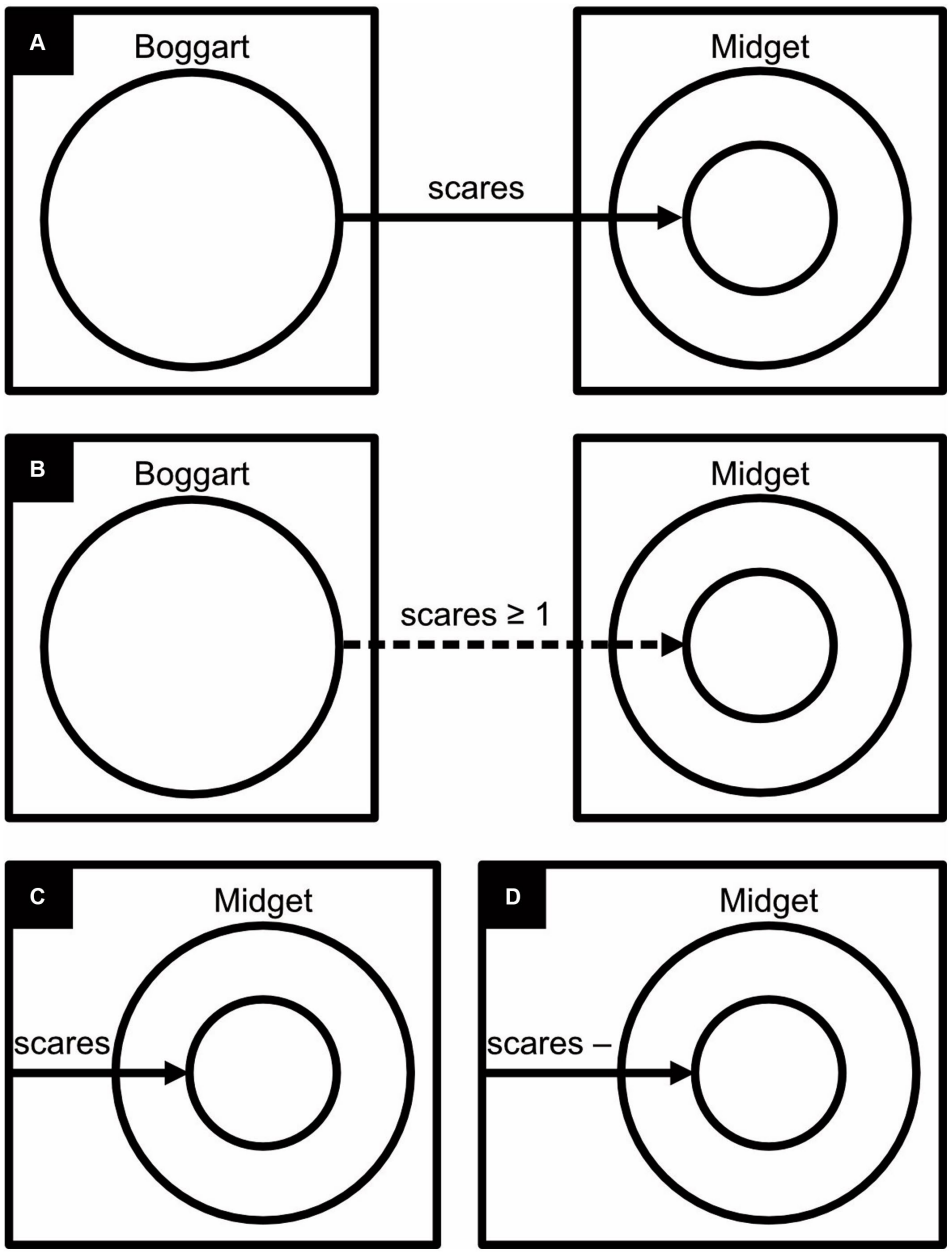
Monochrome concept diagrams. The diagrams express: **(A)** “Boggarts scare only Midgets,” **(B)** “Boggarts scare at least one Midget,” **(C)** “There is something unspecified, and it is the only thing that scares Midgets,” **(D)** “There is something unspecified, and only Midgets scare it”.

**Figure 14:**
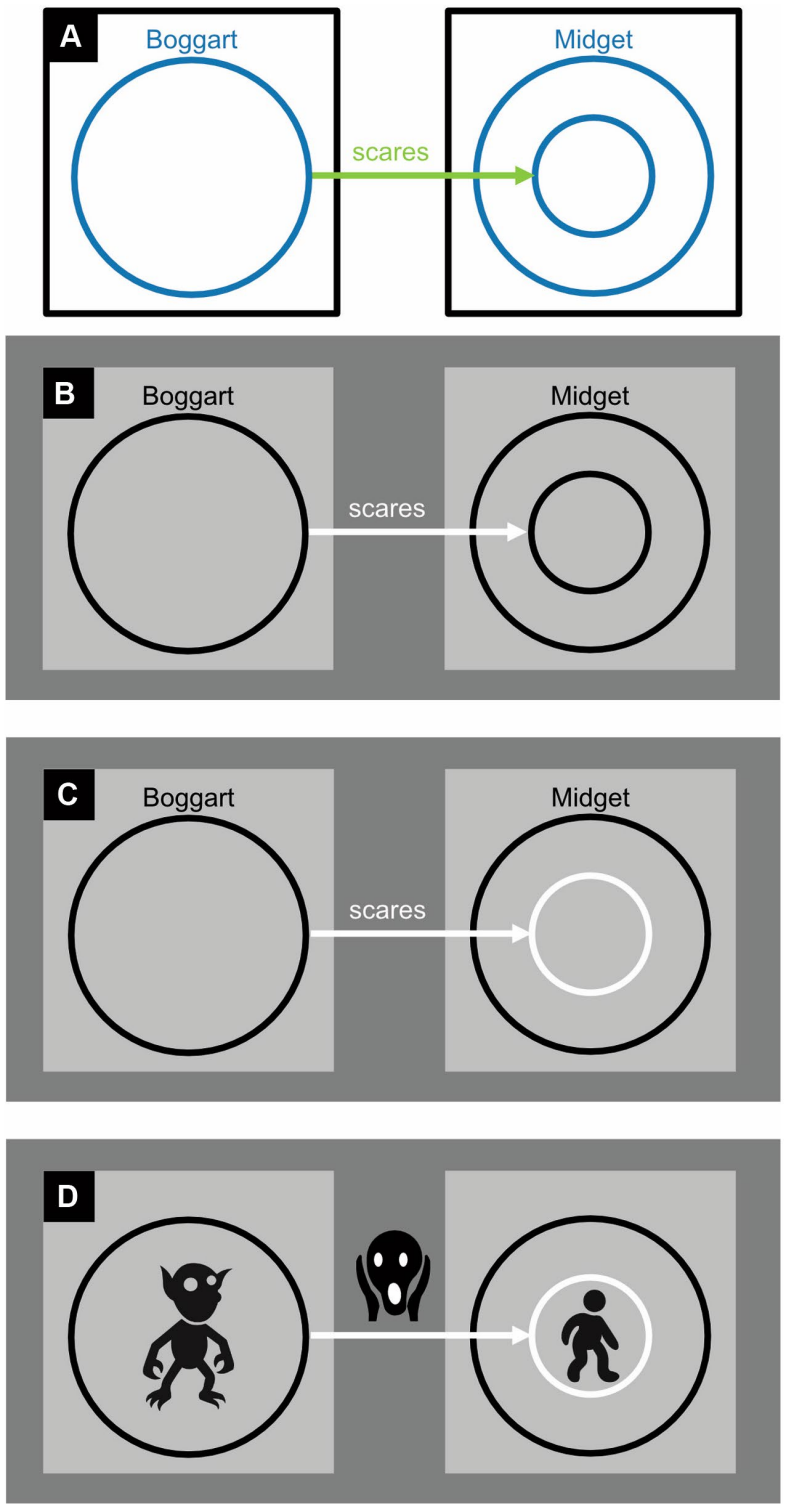
Dichrome concept diagrams: **(A)** To distinguish them better, circles and arrows are colored, respectively, blue and green rather than black. **(B)** Proposal for a monochrome alternative. **(C)** Proposal for a monochrome alternative in which an arrow pointing to a circle is given the same color as the circle itself. **(D)** Replacing abstract labels (here “Boggart,” “scares,” and “Midget”) with concrete icons ought to improve readability. Here, the three icons represent, respectively, a boggart (a short, malicious creature), a facial expression of fear, and a midget (a short but amiable person).

**Figure 15:**
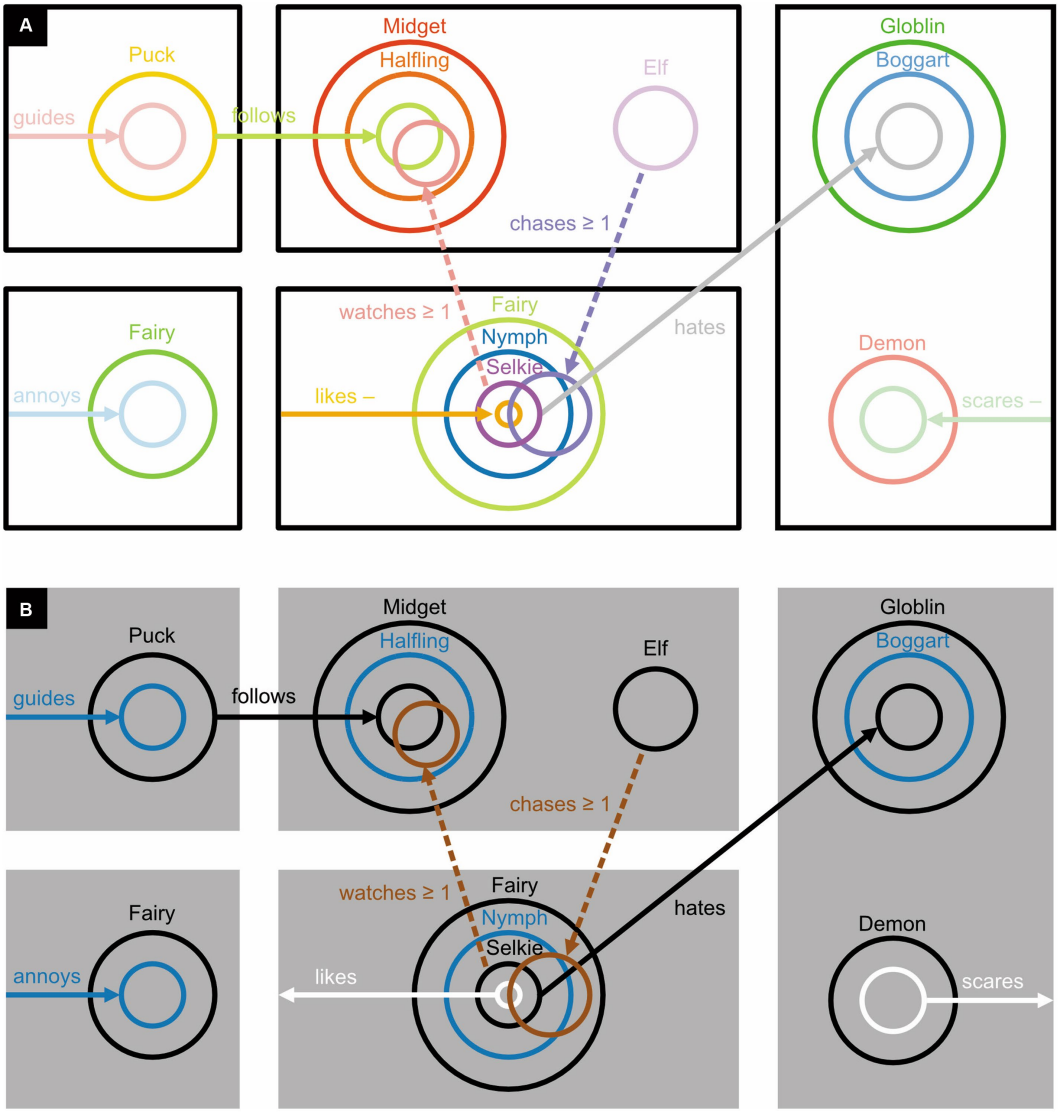
Polychrome concept diagrams. **(A)** Using color to uniquely identify each circle and any associated arrow. **(B)** Using color to make each circle and any associated arrow more distinguishable from nearby or overlapping ones.

McGrath et al. (2022) investigated elaborations of Euler diagrams (Figure 1): *concept diagrams* (Figures 13–15). In concept diagrams, separate Euler diagrams, each represented within a rectangular area, may be interconnected and enriched with extra elements (Figures 13; McGrath et al., 2022). Most of these additions are not very iconic but because the Euler diagrams are, their combination is still fairly iconic.

In examples that refer to mythical creatures, McGrath et al. (2022) added solid arrows to their diagrams to express only-relationships, like in “Boggarts scare only Midgets” (Figure 13A), and dashed arrows to express some-relationships, like in “Boggarts scare some Midgets” (Figure 13B). Mathematical symbols express, say, whether Boggarts scare at least one Midget (Figure 13B). Arrows that start from the edge of a rectangle are used to express that something unspecified is related to something else, like in “There is something unspecified, and it is the only thing that scares Midgets” (Figure 13C). A minus sign inverts a relationship, like in “There is something unspecified, and only Midgets scare it” (Figure 13D).

McGrath et al. (2022) hypothesized that concept diagrams should be easier to read, and work with, if their circles and arrows were colored differently (Figure 14A) or if each circle and any associated arrow could be uniquely identified with a specific color (Figure 15A). Previously, in fact, a similar use of color has been found to improve reasoning with Euler diagrams (Blake et al., 2014). To the authors’ surprise, however, the data did not corroborate their hypothesis. The authors concluded that the more complex concept diagrams are, the less color helps to clarify them.

### 3.2 Using color to better distinguish sets and relationships

McGrath, Howse, and their colleagues should be commended for opening a new, much needed research field; for attempting to render logic more ergonomic; and for letting data rather than intuition be the arbiter of this endeavor’s success. Their experimental studies also suggest, however, that it would have been desirable to get behavioral scientists involved. To an expert in color perception, for example, it is immediately apparent that McGrath et al. (2022)’s study is confounded and its conclusion premature.

The dichrome and polychrome conditions featured colored circles. Unfortunately, the Ishihara colorblindness test (Ishihara, 1917), although quick and easy to perform, was not used to screen out colorblind participants. More importantly, in the dichrome and polychrome conditions, the colored circles and arrows were more luminant (physically lighter) than the black ones in the monochrome condition. As a result, they contrasted less well with their white background and were therefore less visible. Moreover, the dichrome condition (Figure 14A) did not use complementary colors like blue and yellow or red and green, which are the easiest to tell apart, but blue and green. These blue and green colors also had rather similar luminances, which further reduced their distinguishability. In simple diagrams like Figure 14A distinguishability may not be a major issue but it is in busy ones like Figure 15A (Manassi and Whitney, 2018; Wolfe et al., 2022; see also Alqadah et al., 2016). And whereas achromatic colors like black, white, and gray can be perceived everywhere in the eye, chromatic ones like blue and green are only perceived in its central part—the fovea. In sum, the visibility of the circles and arrows was bound to be much better in the monochrome condition (Figure 13) than in the dichrome (Figure 14A), and especially the polychrome (Figure 15A), condition.

In the dichrome condition, there is actually no need to complicate matters with chromatic colors: achromatic ones will do (Figure 14B). If the arrows and circles are, respectively, represented in white and black on a gray background, or vice versa, they will become particularly easy to distinguish (Masin, 2003; see also Bressan and Kramer, 2008, 2017). If there is just one arrow pointing to each circle, one can make visually obvious to which circle the arrow is pointing by giving both the same color (Figure 14C; see also Figure 15). Readers that are unfamiliar with ancient folklore can use a dictionary to find out what, say, a boggart is but may then still have a hard time picturing one in their mind. Replacing abstract labels like “Boggart” with iconic alternatives solves this problem (Figure 14D) and could also bring other diagrams to life.

Even for normally sighted individuals, the cacophony of colors used in Figure 15A may perhaps be a bit overwhelming. It might have been better to use only colors like blue and yellow and black and white that are easy to distinguish for both normally sighted and colorblind individuals, and to use these colors not to uniquely identify circles but to make nearby and overlapping ones easier to distinguish from one another (Figure 15B). One could depict sets and their relationships in dark colors (here black, blue, and the dark yellow known as “brown”); and sets involved in inverse relationships, and these inverse relationships themselves, in light colors (here white). To depict an inverse relationship, one could, in addition, use an arrow that points away from, rather than toward, its associated circle. The less iconic minus sign could then be dropped.

For the design of their experiment, McGrath et al. (2022) made various other suboptimal choices. Reaction time, for example, was measured without ensuring that the participants responded as fast as possible while maintaining near-perfect accuracy. This goes against the tried and tested method for measuring reaction times and makes the results harder to interpret. More importantly, different participants were enrolled in different experimental conditions (between-subjects design), whereas it would have been better if each participant had been enrolled in all conditions (within-subjects design) with the order of the conditions systematically varied between participants (counterbalanced). Using a between, rather than a within, subjects design is especially a concern in color perception, because even such simple colors as black, white, and gray depend in complex ways on the circumstances under which they are perceived (Bressan, 2006, 2007; Gilchrist, 2006; Kingdom, 2011; Soranzo and Gilchrist, 2019; Murray, 2021; Bressan and Kramer, 2021b). Consider, for example, how the moon looks shiny and almost white in the night’s sky yet dull and dark gray under an astronaut’s white boot (Bressan, 2005; Bressan and Kramer, 2021b).

Imitating the night’s sky, investigators of the perception of black, white, and gray often perform their experiments in a dark laboratory and the most careful among them paint their laboratory black and encase all their equipment in black as well (e.g., Bressan and Actis-Grosso, 2006; see also Gilchrist, 2006). Such stringent control over experimental conditions is most likely unnecessary for studies like McGrath et al. (2022)’s. Yet, McGrath et al. went to the other extreme and allowed viewing conditions to differ from one participant to the next even though this adversely affects a between-subject’s experiment more than it does a within-subjects one. That virtually no significant results were obtained is unsurprising. Thus, whether a judicious use of color can, or cannot, improve the readability of busy concept diagrams remains an open question.

## 4 Profiling talent and disability in logic

### 4.1 It is not truth and falsehood, but death and survival, that reign supreme

Expertise in perception can help us improve the ergonomic features of logic. Expertise in cognition can help us understand who, from this endeavor, is likely to benefit the most. It is important to realize that reasoning logically and thinking straight are two different things. For example, thinking, unlike reasoning, requires picking the right premises to begin with and framing the problem in the right way. In Bohr’s quantum theory of physics, a miniscule particle can be at more than one place at the same time. If one reasons logically from the premise that we can travel back and forth in the three dimensions of space but only forward and never backward in the dimension of time, quantum theory makes little sense. To understand this theory, we need to jettison our preconceived notion of what our world is like.

Our notion of what the world is like is shaped by evolution, and in evolution, it is not truth and falsehood that reign supreme but death and survival (Hoffmann 2015; see also Wood, 2023). Indeed, only a minority of undergraduate students perform well when tested on the Wason selection task (Figure 5)—a task specifically designed to test people’s reasoning skill (Wason, 1968, 2013). We are a social species, however, and most humans are keen detectors of who is violating our social norms (Cosmides and Tooby, 1992; Cosmides et al., 2010). Accuracy in the Wason selection task is, in fact, significantly higher if the very same argument is presented as a moral issue (e.g., about whether babies should drink alcohol or not; Figure 5) rather than as one having merely to do with facts (Fiddick et al., 2017).

Logical reasoning and cheater detection can, however, be pitted against each other. Consider these two rules:

Rule 1: If you give me your watch, I give you $20.

Rule 2: If I give you $20, you give me your watch.

Now, if I do not give you $20, Rule 1 implies that you do not give me your watch. Yet, from a logical point of view, Rule 2 does allow me to keep my $20 and still take your watch. This violates the rules of ethics but to the confusion of many socially sensitive people, it does not violate the rules of logic (Cosmides and Tooby, 1992). Teaching aspiring logicians to disregard the content of an argument, and simply and rigidly apply the rules of logic, may improve these people’s reasoning. The issue discussed here, however, brings us to a theory of the development of social and technical skill that has direct implications for how logic could become more ergonomic.

### 4.2 Symbols are for nerds, icons for everybody

The *diametric theory of genomic imprinting* suggests that there tends to be a tradeoff between social skill and mathematical (and thus also logical) ability (see Bressan and Kramer, 2021a and references therein, including especially Crespi and Badcock, 2008; Badcock, 2009, 2019; see also Úbeda, 2008; Del Giudice et al., 2010; Úbeda and Gardner, 2015; Mokkonen et al., 2018; Crespi, 2020; Kramer, 2022). Tellingly, indeed, colloquial language has one single term for a person who is both socially awkward and has an all-absorbing attention for the minute details of some technical matter: not always derogatively, a person like that is called a *nerd*.

Now, some of the genes we inherit from our parents are turned on and some off. Remarkably, of so-called *imprinted genes*, those turned on in the copy we inherit from our father are typically turned off in the copy we inherit from our mother and vice versa. As a consequence, fathers and mothers push offspring development in diametrically opposite directions. To some extent, male and female sex chromosomes have similar opposite effects (Crespi and Badcock, 2008; Badcock, 2009, 2019).

The root of the parental conflict has to do with the fact that, especially during gestation and the first few years of the offspring’s life, mothers invest more in their children than fathers do (Moore and Haig, 1991; Haig, 2010; Kotler and Haig, 2018). For the most part, paternal imprinting pushes for the extraction of maternal resources during this early period and promotes the growth of the offspring’s body and brain—in particular those parts of the brain that allow it to deal with its physical environment. Maternal imprinting, instead, limits the growth and resource extraction, but does stimulate the growth of those parts of the brain that allow the offspring to deal with its social environment—which, later on, will enable it to take its mother’s directions.

The tug of war between parents over the expression of genes goes at their offspring’s expense, and imprinted genes and sex chromosomes are implicated disproportionately often in both physical and mental disease—most prominently in pairs of diseases that are due to tightly related genetic mutations that come with roughly opposite symptoms (Xirocostas et al., 2020; Bressan and Kramer, 2021a). Excessive paternal imprinting is associated with overgrowth of body and brain before birth, which makes delivery riskier for the mother (see also Kramer and Bressan, 2019). In addition, it is associated with autism-spectrum disorders and strong semantic memory for facts (including technical ones) but weak episodic memory for events (including social ones). Conversely, excessive maternal imprinting is associated with undergrowth of body and brain before birth, psychosis-spectrum disorders, and strong episodic but weak semantic memory (Bressan and Kramer, 2021a). A slight tendency toward autism is associated with increased attention to detail, strong semantic memory, and increased technical and mathematical ability but reduced episodic memory and social skill, including a diminished ability to understand other people and to detect whether they are cheating or not; a slight tendency toward psychosis is associated with the opposite (Crespi and Badcock, 2008; Badcock, 2009, 2019; Bressan, 2018; Kramer, 2022 and references therein).

Malnutrition, brain damage, and problems during development may all result in memory deficits. Yet, if the diametric theory of genomic imprinting is correct, then many people who are healthy and have developed normally, but in whom maternal imprinting happens to be dominant, can have a weak semantic memory too. These days, the less people are inclined to think analytically, the more they rely on the internet as a kind of external semantic memory (Barr et al., 2015). Hotly debated is whether this may further weaken internal semantic memory and its cortical substrates (Firth et al., 2019; see also Dempsey et al., 2019; Ellis, 2019; Hartanto et al., 2023). So, if we wish to improve reasoning in not just professional logicians but other people too, we ought to introduce a tool that requires as little as possible from our semantic memory. Arguing for a more ergonomic representation of knowledge, Wood (2023, p. 1) observes that “if knowledge were simpler, we would all be wiser.” Likewise, it stands to reason that if logic burdened semantic memory less, we would all argue better. And as icons are better mnemonics than symbols are (Kramer, 2023), iconic logic is more ergonomic than symbolic logic is. Although strong analytical thinkers may certainly benefit from iconic over symbolic logic, those who need a reasoning tool the most ought to benefit the most too.

### 4.3 Put not scribes, but readers, first

Given that symbolic logic burdens its readers’ semantic memory more than does iconic logic, one may wonder why most logicians stick to symbolic logic anyway. Excerpts from the medical history of a representative logician (Charles Sanders Peirce) may help answer this question (Pfeifer, 2014). There is little doubt Peirce paid close attention to detail and was exceptionally well-versed in the slow, painstaking, step-by-step kind of thinking that comes in handy in formal logic. The excerpts suggest, however, that Peirce did not consider himself a good writer, was not in tune with his audience during lectures, and had “too little social talent, too little art in making himself agreeable” (Pfeifer, 2014, p. 29).

The combination of a poor ability to put oneself in the mind of another person, and poor social and communication skills, but keen attention to detail, and a strong tendency to systematize, is typical of people with an autistic tendency and frequent among mathematicians (Crespi and Badcock, 2008; Badcock, 2009, 2019; Bressan, 2018; Bressan and Kramer, 2021a). Autism is partly heritable (Badcock, 2009, 2019), and Peirce came from a math-oriented family (Pfeifer, 2014). It is thus tempting to suspect Peirce had an autistic tendency (for a similar view, see Pfeifer, 2013; for whether Peirce may have had additional bipolar tendencies, see Brent, 1998).

If Peirce did indeed have an autistic tendency, he may not have understood how unergonomic it is to represent two distinct items with one and the same line of identity while, under circumstances, allowing one and the same item to be represented with two distinct lines. He may not have understood that it is easier to see whether a region is shaded or not than to have to count to see whether it is oddly or evenly enclosed. And he may not have understood that the meaning of labels of items or properties are easier to picture and remember when they are expressed with concrete icons than when they are expressed with abstract letters or words (Kramer, 2023). Peirce deemed the shading of regions, and presumably also the drawing of little pictures, too much of a hassle for scribes. Yet with readers typically far outnumbering scribes, one would think that reducing the burden on all these readers should count much more than reducing any burden on the scribes (see also Kramer, 2023). Today’s software lightens the scribe’s task enormously, and there is now all the more reason to accommodate the reader.

The suggestions for improvement of the ergonomic features of iconic logic put forth in this article, of course, require further investigation and especially empirical testing. The purpose of this paper is not to give the last word on any empirical matter but rather to show behavioral scientists an opportunity for conducting behavioral research in an area of scholarship (formal logic) that at first sight does not seem offer such opportunities. Conversely, it is also meant to encourage logicians to improve the ergonomic aspects of their logical systems and to convince those few that conduct behavioral experiments for this purpose to take advantage of the expertise of behavioral scientists. Like mathematics, logic has traditionally been viewed as standing apart from the empirical sciences. Yet, to better serve its users, it is high time that, for its ergonomic aspects, logic becomes an empirical science too.^2^

## Author contributions

PK: Writing – review & editing, Writing – original draft.

## Footnotes

^1^Peirce did allow the overlapping of the boundaries of regions, but this is an unnecessary complication (Dau, 2006).

^2^For a similar argument for mathematics more generally, see (Kramer, 2022, #269048).
